# Testing bio-efficacy of insecticide-treated nets with fewer mosquitoes for enhanced malaria control

**DOI:** 10.1038/s41598-018-34979-3

**Published:** 2018-11-13

**Authors:** Sebastien Boyer, Emilie Pothin, Sanjiarizaha Randriamaherijaona, Christophe Rogier, Thomas Kesteman

**Affiliations:** 1 Unité d’Entomologie Médicale, Institut Pasteur de Madagascar, BP 1274, Avaradoha, Antananarivo 101 Madagascar; 2 Medical Entomology Platform, Institut Pasteur du Cambodge, 5 Boulevard Monivong, Phnom Penh, Cambodia; 3 Department of Epidemiology and Public Health, Swiss Tropical and Public Health Institute, Basel, Switzerland; 4 Ecole doctorale Sciences de la vie et de l’environnement, Université d’Antananarivo, Antananarivo, 101 Madagascar; 5 Malaria Research Unit, Institut Pasteur de Madagascar, BP 1274 Avaradoha, Antananarivo, 101 Madagascar; 6 Unité de recherche sur les maladies infectieuses et tropicales émergentes (URMITE) - UMR 6236, 27 boulevard Jean Moulin, 13385 Marseille Cedex 05, France; 7 Institute for Biomedical Research of the French Armed Forces (IRBA), BP 73, 91223 Brétigny-Sur-Orge Cedex, France; 8 Fondation Mérieux, 17 rue Bourgelat, 69002 Lyon, France

## Abstract

Malaria control programs implementing Long-Lasting Insecticidal Nets (LLINs) are encouraged to conduct field monitoring of nets’ survival, fabric integrity and insecticidal bio-efficacy. The reference method for testing the insecticide activity of LLINs needs 100 two-to-five-day-old female mosquitoes per net, which is highly resource-intensive. We aimed at identifying an alternative protocol, using fewer mosquitos, while ensuring a precision in the main indicator of ±5 percentage points (pp). We compared different laboratory methods against the probability of the LLIN to fail the test as determined by a hierarchical Bayesian model. When using 50 mosquitoes per LLIN and considering mortality only instead of mortality or knock-down as validity criteria, the average error in the measure of the proportion of nets considered as valid was 0.40 pp. The 95% confidence interval of this value never exceed 5 pp when the number of LLIN tested was ≥40. This method slightly outperforms the current recommendations. As a conclusion, testing the bio-efficacy of LLINs with half as many mosquitoes provides a valid evaluation of the proportion of valid LLINs. This approach could increase entomology labs’ testing capacity and decrease costs, with no impact in the decision process for public health purposes.

## Introduction

Mass distribution of Long Lasting Insecticidal Nets (LLIN) is the vector control intervention most used against malaria in sub-Saharan Africa, covering 44% of the population at risk, far ahead of indoor residual spraying, which covers only 7%^1^. The intensification of vector control has led to an important decrease of malaria cases in the last decade. Recent studies confirmed that this significant decline in mortality is due mainly to the use of insecticide-treated nets, which both physically isolate people from vectors, and reduce the life span of vectors^2^. However, malaria still kills about 500,000 people a year, with 198 million cases reported in 2013^1^.

In a global context of plateauing international funding^1^, malaria control programs are asked to improve their performance with a constant budget. In the absence of groundbreaking new control tools, the assessment of current control measures is paramount. The entomological effectiveness of vector control interventions relies on the overall quality of the intervention (e.g., bed net durability and proper use), the susceptibility of vectors to insecticides, and on the biting behavior of vectors^3,4^. Changes to any of these three parameters are expected to affect the operational bio-efficacy of LLINs. Malaria control programs and donors thus more and more want to test the LLINs for durability and insecticide bio-efficacy, in order to evaluate their effectiveness, before and after their distribution to the population.

The World Health Organization’s Pesticide Evaluation Scheme (WHOPES) therefore published detailed recommendations on how to perform testing of the insecticide bio-efficacy of LLINs. These guidelines give a precise description of the assessment method for individual nets in the context of phase III field trials, although the rationale for this methodology is not presented. The operator should cut equal areas from all five sides of each net in predetermined positions, and then expose each piece to 20 mosquitoes distributed among four standard WHO cones^3^. The LLIN is considered as valid if ≥80% mosquitoes are dead after 24 hours or if ≥95% mosquitoes are knocked down (KD) after 1 hour^3^. A batch of nets (30–50 nets per brand/product) passes the phase III if 80% of the batch are valid 3 years after distribution. Unfortunately, these guidelines provide no guidance for the interpretation of this key indicator - the proportion of valid LLINs - for observational effectiveness studies (phase IV).

Overall, a LLIN needs 100 susceptible, two- to five-day-old female mosquitoes to be tested. Applying this protocol to a large number of LLINs requires considerable logistics and a high-performance entomology lab in order to breed and grow enough mosquitoes. For example, an experiment conducted in Madagascar required the breeding of about 50,000 two-day-old females in order to test 400 treated nets, i.e., approximately 30 nets per batch, considering 3 brands, at 3 time points, in six locations and untreated nets used as negative control^5^. This study required a fully dedicated entomology team to work continuously for 24 months, considering a daily production of about 300 females a day^5^. Most countries affected by malaria can receive millions of LLINs each year but are highly constrained in financial, logistical, and trained human resources for entomological investigations. If the number of mosquitoes needed to test the bio-efficacy of LLINs could be reduced, the human and animal resources, the duration, and the costs of an evaluation would be reduced in equal proportion.

This paper explores the consequences of reducing the number of mosquitoes used in the assessment of the validity of LLINS. To fulfill this objective, two approaches were considered: (i) a preliminary theoretical analysis of the effect of the number of mosquitoes used on the risk of misclassification of a single net as valid or invalid, and (ii) an empirical analysis using a Bayesian model to assess the effect of reducing the number of mosquitoes on the performance of the tests, and in particular on the measured proportion of valid LLINs, which is the key indicator for decision-making in public health.

The preliminary analysis compared the risk of misclassifying a net, i.e., passing an invalid net or failing a valid net, for different experimental protocols using one, two, three or four cones per LLIN side. Using a simplistic approach with binomial distributions for both mortality and knock-down rates we estimated the range in which the confidence of the test results would be poor, arbitrarily defined as the “danger zone” in which the risk of erroneous classification is >10%.

A second analysis aimed at exploring what performance would be achieved while testing with a different number of cones/mosquitoes. Therefore, we evaluated the performance of 12 scenarios: testing LLIN insecticide efficacy with one, two, three or four cones per LLIN side, against three different outcomes, i.e., the outcome proposed in WHOPES guidelines (≥80% mortality or ≥95% KD), mortality only (≥80%), and KD only (≥95%). Given the lack of a gold standard, the performance of the tests for each scenario were assessed by comparing results against the probability of a net being valid, estimated using a hierarchical Bayesian model fitted to the full set of data for 235 LLINs of diverse ages (new, 6, or 12 months-old), collected from communities in Madagascar, and evaluated using the full WHOPES procedure. We used a resampling approach to test the effect of reducing the number of cones/mosquitoes included in the analysis. Using the full set of nets, we first assessed the validity of each net (expressed as a probability), then for each sub-sample of nets, we compared results between measured outcomes and estimates from the reference (Bayesian) model to derive the test performances of each of the 12 scenarios. The main performance criterion was the precision in the measure of the proportion of valid LLINs, the key indicator for public health decisions. We considered that the measured proportion of valid LLINs should not deviate from the model estimate by more than 5 percentage points (pp) in 95% of the samples. Therefore, an important feature of each scenario is the minimum sample size (number of LLINs) required to ensure that the 95% confidence interval (CI) around the measure of the overall proportion of valid LLINs is included within the interval [estimate from the Bayesian model ±5 pp].

## Results

**Figure 1:**
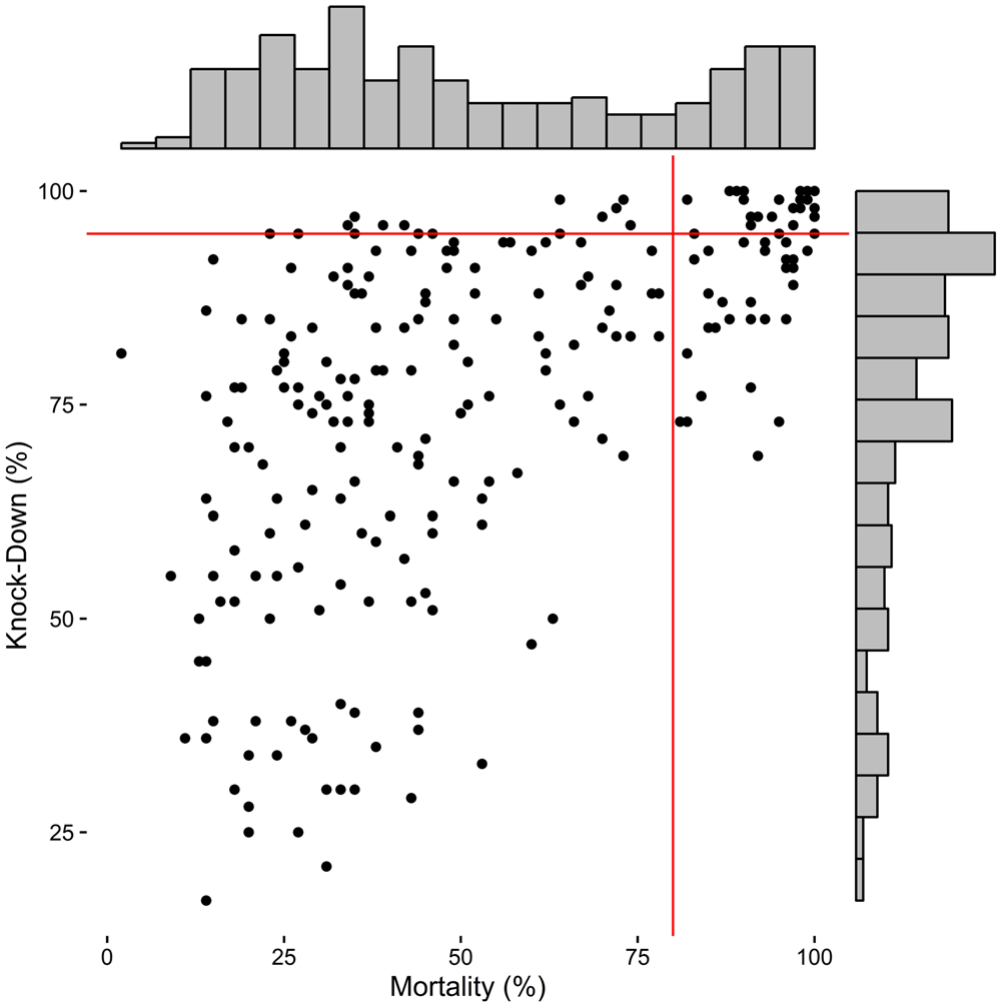
Scatterplot and histograms of mortality and knock-down (KD) rates for each LLIN as observed with four cones per LLIN. Red lines: WHOPES cutoffs.

In our dataset, considering the WHOPES criteria (i.e., using four cones per LLIN side; ≥80% mortality or ≥95% KD cutoff), 32.8% of the tested LLIN were considered valid. Considering the mortality and the KD criteria separately, 26.4% and 20.4% respectively of the LLINs were considered valid (Fig. 1).

The preliminary analysis of the trade-off between effort (i.e., the number of mosquitoes used by LLIN) and accuracy (i.e., proportion of nets correctly classified) shows that the “danger zones” in which a single LLIN has a high risk (>10%) of being misclassified are similar for four, three or two cones; these zones are broader when only a single cone is considered (Table 1). The range of this danger zone is more than twice as wide (+102%) when only one cone is used instead of four, but it increases by only 42% if two cones are considered.

**Table 1:** Intervals of mortality and knock-down rates in which the risk of wrongly classifying a LLIN is >10%.

Number of cones	Mortality rate		Knock down rate	
	Interval (%)	Range (pp)	Interval (%)	Range (pp)
1	66.0–86.9%	20.8	85.3–97.8%	12.5
2	70.9–85.5%	14.6	89.7–97.8%	8.1
3	72.8–84.7%	11.9	89.6–96.7%	7.1
4	73.9–84.2%	10.3	90.9–96.8%	5.9

**Figure 2:**
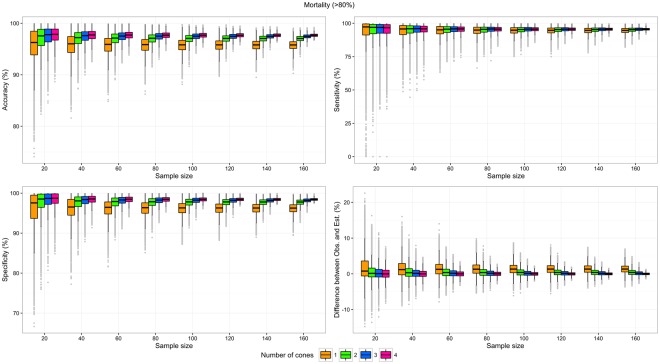
Test characteristics of scenarios testing mortality with one to four cones per LLIN side, by sample size. Upper left: Accuracy; Upper right: Sensitivity; Lower left: Specificity; Lower right: Difference between the proportions of valid LLIN observed with n cones and the proportion of valid LLINs estimated by the Bayesian model.

**Figure 3:**
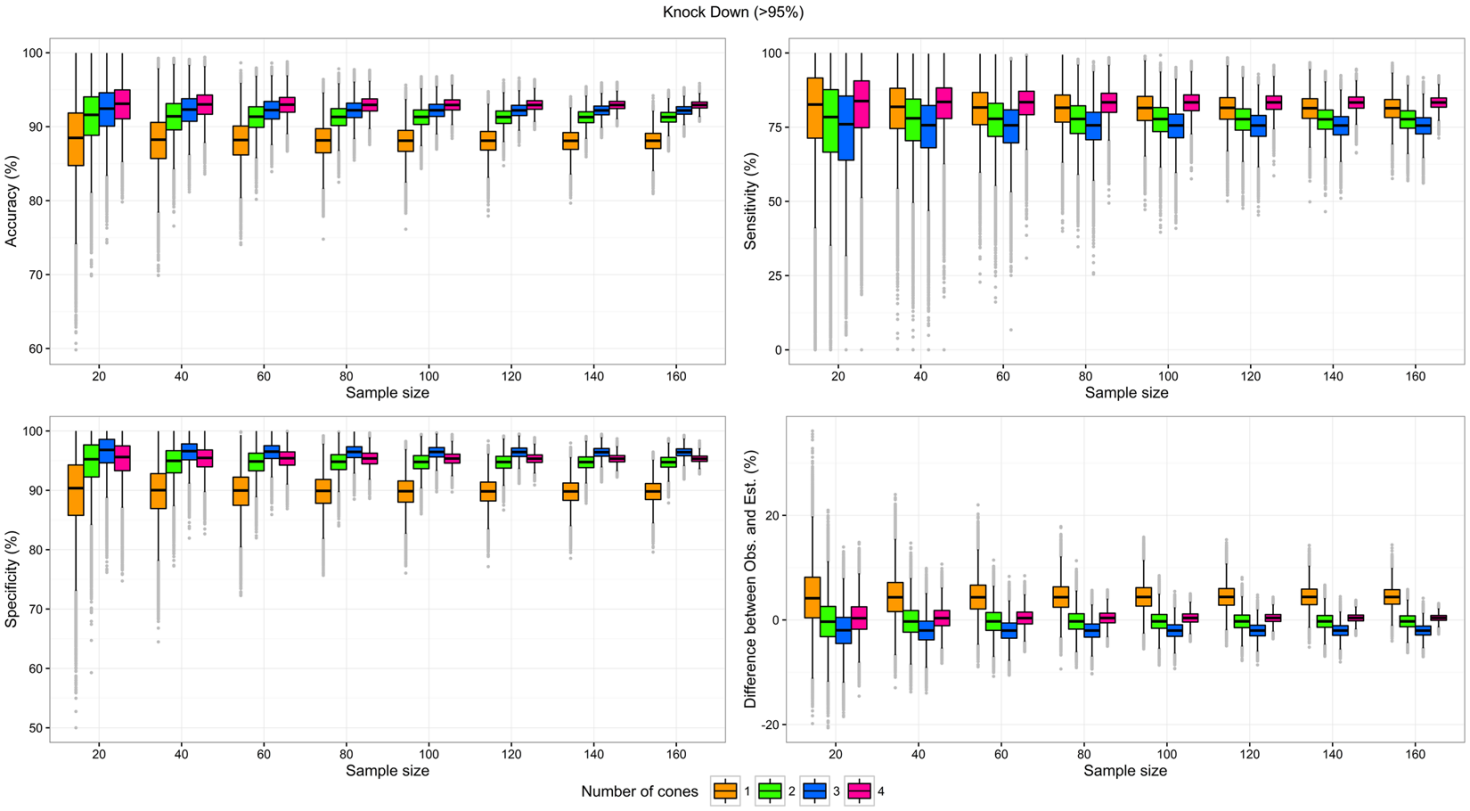
Test characteristics of scenarios testing the Knock-Down effect with one to four cones per LLIN side, by sample size. Upper left: Accuracy; Upper right: Sensitivity; Lower left: Specificity; Lower right: Difference between the proportions of valid LLIN observed with n cones and the proportion of valid LLINs estimated by the Bayesian model.

**Figure 4:**
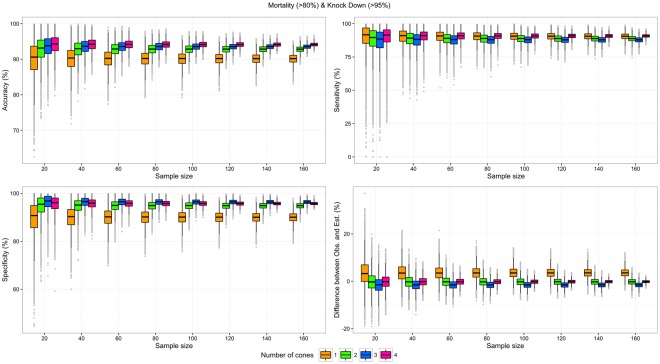
Test characteristics of scenarios testing both mortality and Knock-Down effect with one to four cones per LLIN side, by sample size. Upper left: Accuracy; Upper right: Sensitivity; Lower left: Specificity; Lower right: Difference between the proportions of valid LLIN observed with n cones and the proportion of valid LLINs estimated by the Bayesian model.

Over the 12 scenarios, the average accuracy varied between 88.0% and 97.7%, the average specificity between 89.7% and 98.4%, the average sensitivity between 74.8% and 95.4%, and the average absolute difference between observed and estimated proportion of valid LLINs varied between 0.0 pp and 4.4 pp (Figs 2–4 and Table 2); average refers to the mean throughout the manuscript. The best performances were obtained by scenarios with mortality outcome only while the worst performances were obtained with KD results only.

**Table 2:** Accuracy, sensitivity, and specificity of mortality, knock-down, and WHO’s mixed criterion.

		1 cone	2 cones	3 cones	4 cones
≥80% mortality or ≥95% KD	Accuracy (%)	90.1	92.8	93.5	94.1
	Specificity (%)	89.9	94.7	96.3	95.7
	Sensitivity (%)	90.3	88.5	87.6	90.6
	Difference between estimated and observed (pp)	3.6	−0.2	−1.5	−0.2
	Minimum sample*	160	60	80	40
≥80% mortality	Accuracy (%)	95.7	97.0	97.5	97.7
	Specificity (%)	96.2	97.7	98.1	98.4
	Sensitivity (%)	94.3	94.9	95.3	95.4
	Difference between estimated and observed (pp)	1.4	0.4	0.2	0.0
	Minimum sample*	80	40	<20	<20
≥95% KD	Accuracy (%)	88.0	91.3	92.2	92.9
	Specificity (%)	89.7	94.7	96.3	95.3
	Sensitivity (%)	80.8	77.0	74.8	82.7
	Difference between estimated and observed (pp)	4.4	−0.3	−2.0	0.4
	Minimum sample*	>160	80	120	40

*Minimum sample size required to achieve a precision of 5 percentage points in the measure of the proportion of valid LLIN.

In scenarios using mortality outcome only, sensitivity was ≥94%, specificity ≥96%, and accuracy ≥95%. These characteristics were much more variable in scenarios using the KD outcome, where the sensitivity varied between 74.8% and 82.7%, and the specificity between 89.7% and 96.3%. Similarly, in scenarios using the mixed outcome the sensitivity varied between 87.6% and 90.6% and specificity varied between 89.9% and 96.3%.

The difference between estimated (model) and observed proportions of valid LLINs was the main indicator from the empirical analysis. Unsurprisingly, these differences were very similar (range: −2.0 pp to 4.4 pp) on average, but their confidence intervals were broader as the number of LLIN tested and the number of cones used decreased (Figs 2–4). Importantly, the minimum samples needed to obtain a precision of ±5 pp in the measure of the proportion of valid LLIN were much higher for one-cone scenarios (range: 80 to >160 LLINs) than for other scenarios, especially those using two or four cones (range: <20 to 80 LLINs). These minimum samples were the lowest with scenarios using the mortality as the unique validity criterion, half of those in corresponding scenarios using combined mortality or KD validity criteria. Three of the scenarios using the mortality as the unique validity criterion (with two, three, and four cones) demonstrated a minimum sample size ≤40 LLINs.

The difference between the proportion of LLINs defined as valid based on testing two cones per side of LLIN and the proportion calculated by the Bayesian model was <1 pp on average, for the three outcomes considered, and the minimum samples to achieve a precision of ±5 pp were ≤80 LLINs. With a sample size of 40 LLINs, and considering the mortality only, the 95% CI of the error in the measure of the proportion of valid LLIN was [−2.95; 4.07]. This interval is narrower than the 95% CI of the error in the measure of the proportion of valid LLINs obtained with the same sample size, but considering mortality and KD, as recommended by WHOPES, which was [−4.01,3.69] (Annex 2).

## Discussion

The results showed that using only two cones per side of LLIN instead of four, and considering mortality only instead of mortality or KD as validity criteria, the mean error in the measure of the proportion of nets considered as valid was less than 0.5 pp, and when testing at least 40 LLINs, it is very unlikely that this indicator deviates more than 5 pp from its expected value (i.e., its 95% CI is below ±5 pp). This precision is very similar to, and even slightly better than, what is achieved when applying WHOPES criteria (mortality or KD) on the same sample size. Halving the numbers of cones per each LLIN side for the evaluation of the bio-efficacy of LLINs is thus possible and induces insignificant bias in comparison with the estimated and observed four-cone results. The error in the main estimate would almost never exceed 5 pp, which is acceptable for decision in public health since one can consider the bio-efficacy of LLINs either as preserved (e.g., >80% valid), reduced (e.g., 61–80%), seriously affected (e.g., 31–60%), or insufficient (e.g., ≤30%) according to the mortality rate. In fact, the proportion of valid LLINs is the only indicator that will subsequently be used for public health decision.

With this method, a sample size of 40 nets or more is needed to ensure that the 95% CI of the error in the measure of the proportion of valid LLIN does not exceed 5 pp, whether using mortality only, or mortality and KD as outcomes. This result confirms that the minimum sample size recommended by WHOPES (30–50 nets) is reasonable, and validates *a posteriori* our methodology.

The results of the empirical part of the study are consistent with the preliminary analysis that showed how the limits of the “danger zone” remains very similar for two, three, and four cones for both mortality and KD. Thus, while still accounting for variability due to nets, facets and cones, the results still hold independently of performances of the nets. The WHOPES criteria are therefore conservative enough that decreasing the number of mosquitoes does not affect much the confidence of the test.

The two main outcomes proposed in WHOPES guidelines to assess the bio-efficacy of LLIN on mosquitoes exposed during three minutes are: (i) a mortality after 24 hours ≥80%, and (ii) a KD rate after 60 minutes ≥95%. The standard protocol recommends using a mixed outcome, i.e., mortality 80% or KD ≥ 95%. Results showed that mortality outcome was better than both KD and mixed model outcomes at predicting the validity of LLINs with fewer mosquitoes. Moreover, new generation insecticides have a delayed mortality effect, but no KD effect^4^. Our findings advocate for focusing on mosquitoes’ mortality in the evaluation of the bio-efficacy of LLINs, as suggested by other authors^6–9^.

A major limitation of the present study is that a single database of LLINs has been used, all coming from the same country. This could limit the external validity of our study, e.g., performance would be poorer if mortalities (or KD rate) were distributed closer to the threshold. Three factors mitigate this limitation. First, as observed in Fig. 1, mortality and KD rates both display broad distributions. In contrast to very replicable results that would be expected if most LLINs exhibited either 0% or 100% mortality (and KD), our sample exhibited mortality rates across the possible range. This is likely attributable to the different ages (new, 6 and 12 months-old), different trademarks (n = 3, Annex 1), and different places of distribution (n = 6) -and thus variety of use^10^ of the nets. In general, field conditions are likely to be more homogeneous than those of our dataset since net distributions are usually uniform in the trademark and insecticide, and batches of nets tested together are usually of the same age. Since our results were drawn from a diverse sample of nets, it seems likely that our conclusions also apply to situations where the sample of nets is more uniform. The second reason is that the theoretical analysis of the trade-off between effort and performance already predicted that the use of only two cones should affect marginally the performance of the test as compared with four cones. The last factor that mitigates the limitation imposed by the analysis of a single database is that we analyzed a large number of iterations, thus covering a very wide range of scenarios, including extreme ones. The most extreme scenarios can be performed by the observation of the results beyond the 95% CI. Results of the 99% CI also showed that there is only a few pp difference between the uses of two or four cones (Annex 2). For example, the 99% CI of the precision in the measure of the proportion of valid LLINs, based on WHOPES’ mixed criterion and for a sample of 60 LLINs, is [−6.00, 5.88] for two cones versus [−4.05, 3.75] for four cones, and even more similar CI with mortality outcome only: [−3.26, 4.43] vs. [−2.13, 2.08]. These differences are not significant for public health decisions.

The present study shows that using 50% fewer mosquitoes on a sample of 60 LLINs or more has a negligible impact on the assessment of the validity of mosquito nets according to mortality criteria in bio-efficacy assay, and no impact on the process of decision for public health purposes. It demonstrates that it is possible to reduce the number of mosquitoes bred, the number of cages and bins, the amount of food for larvae, the technician’s time needed for raising and breeding mosquitoes, bioassay duration, and therefore the overall cost and time for LLIN testing. These results should open the discussion among researchers and guideline-writers about the current WHOPES method for evaluating LLIN bio-efficacy.

## Methods

### Sampling of LLIN

The study sites selected for this study represented some of the environment and cultural settings in which LLINs are distributed. A cross-sectional household survey was conducted in four regions of Madagascar (Antsiranana I, Antsiranana II, Morondava, Toamasina). The nets were impregnated either with α-cypermethrin (one brand) or deltamethrin (two brands).

### Reference sensitive strain

An *Anopheles arabiensis* sensitive strain has been grown at the Institut Pasteur de Madagascar since April 2010^11^. This colony was characterized in the laboratory for insecticide susceptibility using standard WHO impregnated paper tests: 100% mortality was observed with DDT (4%), fenithrotion (1%), propoxur (0.1%), permethrin (0.75%), deltamethrin (0.05%) and bendiocarb (0.1%).

A hundred mosquitoes were tested for the presence of resistance genes by PCR assays. Each mosquito was extracted using two or three legs following the protocol described by Cornel and Collins^12^. Leg extractions were used to genotype samples for the *Kdr* allele, using a PCR diagnostic test for detection of *Kdr* “Leu-Phe” mutations following the protocol described by Martinez-Torres *et al*.^13^. Mosquitoes were screened for insensitive acetylcholinesterase (*Ace-1*) by the PCR method of Weill *et al*.^14^. No *Kdr* or *Ace-1* resistance alleles were detected.

### Insecticidal activity - WHO cone bioassays

**Figure 5:**
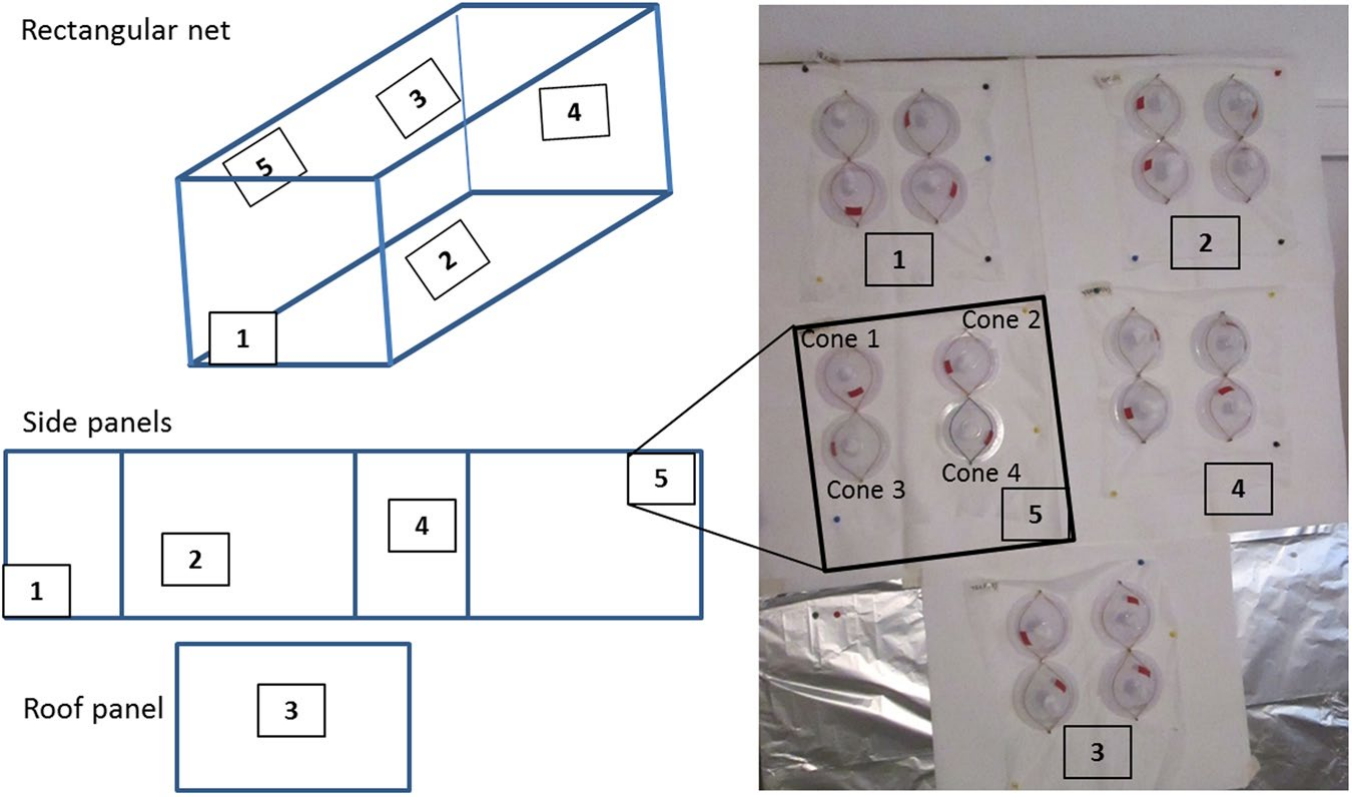
Recommended positions from which netting pieces should be taken and cone bioassay (WHOPES, 2013). For each LLIN, five net pieces are cut and tested with four cones, each containing five female mosquitoes.

Standard WHO cone bioassays were performed with an *An*. *arabiensis* sensitive strain to determine the bio-efficacy of LLINs as recommended^3^. For each LLIN, five sub-samples were prepared for cone tests, corresponding to the top and the four sides of the net (Fig. 5). Each sub-sample was folded in aluminum foil, labeled, and kept individually in a +4 °C refrigerator prior to assays.

For each individual LLIN sub-sample, four cone tests were conducted at a time following standard WHO procedure^5^. Five non-blood-fed, two-to-five-day-old female *An*. *arabiensis* were introduced into each cone and exposed to LLIN samples for three minutes, before being transferred to paper cups covered with neutral netting, and held for 24 hours at 28 °C and 80% humidity with access to 10% sugar solution. The holding board was held slanted at 90°. Knock-down (KD) was recorded 60 minutes post-exposure and mortality was recorded 24 hours post-exposure. A total of 100 mosquitoes were used to complete a test for each net. Each day of testing, four cones with 10 mosquitoes were fixed on a non-impregnated net for negative control, thus requiring 40 additional female mosquitoes per day.

The first outcome for considering a LLIN as valid was an average mortality of mosquitoes over all cones after 24 hours ≥80%. The second outcome for considering a LLIN as valid was an average KD over all cones after 1 hour ≥95%. The third outcome was the one proposed in WHOPES guidelines and considered a LLIN as valid if the average mortality of mosquitoes over all cones after 24 hours is ≥80% or the average KD over all cones after one hour is ≥95%^3^.

### Trade-off between efforts and performance

A script (Annex 3) was generated on R software in order to find lower and upper limits of the interval (“danger zone”) in which a net would have a probability of >10% to be erroneously misclassified, i.e., either classified as good (mortality/KD rate ≥cutoff) while the net is actually bad (mortality/KD rate <cutoff), or classified as bad while the net is actually good. The range is the difference in percentage points between upper and lower limits of this confidence interval. This calculation considers a plain binomial distribution, and therefore does not account for the variability between nets, between sides and between cones.

### Data analysis

A hierarchical Bayesian model was used to estimate for each net, *i*, the average knock-down probability, *p*_*i*_, and corresponding average mosquito mortality, *q*_*i*_ (see Annexes 4 and 5), allowing for stochastic variation between nets and between the different sides of the nets. This model provided posterior densities for both *p*_*i*_ and *q*_*i*_, which were analyzed to obtain probabilities for each net that the true effect is less than the required standard cut-off value for each outcome. The probability of the mosquito net failing on the ‘knock down’ criterion was thus the cumulative proportion *Pr*(*p*_*i*_ < 0.95). Similarly, the probability of the mosquito net failing on the criterion of mortality only is given by *Pr*(*q*_*i*_ < 0.80). The standard method for classifying a mosquito net as failing, requires both criteria to be satisfied, and therefore its probability is: *Pr*(*p*_*i*_ < 095 & *q*_*i*_ < 0.80).

### Comparisons of methods to estimate of validity of the nets

In absence of a gold standard methodology, it remains impossible to exactly know the validity of a net nor the mortality and KD rates for each mosquito net. The hierarchical Bayesian model allows for an estimation of mortality and KD rates and therefore provides an estimate of the probability of validity of each net while accounting for the structure of a design (i.e., mosquitoes in different cones from different sides of different LLINs). Variability around the estimates is captured through resampling from a database of empirical results, without necessarily increasing the number of empirical tests.

Multiple methods of evaluation of validity of mosquito nets were studied based on the sample of 235 mosquito nets tested. The objective was to compare the characteristics and performance of the different intensities of sampling the mosquito net population, in comparison to the use of the full available dataset to investigate whether a less resource-demanding technique could be used. The different strategies considered multiple aspects: (1) the outcome itself by considering the different criteria separately and simultaneously, (2) the number of mosquito nets tested (between 20 and 160) and (3) the number of mosquitoes cone used on each side (between 1 and 4 cones). Subsets of the full dataset, corresponding to the different strategies were resampled, and for each LLIN included, all possible combinations of one, two, and three cones out of four for every LLIN side were considered. This represents respectively 1024 (=45, i.e., four possible cones on each of the five LLIN sides), 7776 (=65, i.e., six possible combinations of two cones on each of the five LLIN sides), and 1024 (=45, i.e., four possible combinations of three cones on each of the five LLIN sides) combinations per LLIN for one, two, and three cones out of four. The sampling was repeated for 10,000 iterations. For each iteration N nets were sampled amongst a total of 235 (N representing the sample size studied, varying between 20 and 160), without replacement. For each net a single combination of one, two or three cones was sampled amongst the total of all possible combinations. For each net, in each sample, the mortality and KD rates were calculated and compared with WHOPES guidelines’ validity thresholds.

For each iteration, the following performances of the test were estimated: sensitivity (probability of passing a valid net), specificity (probability of failing an invalid net), accuracy (probability of correctly classifying a net) and absolute difference between the observed proportion of valid nets and the predicted proportion estimated from the Bayesian model. To our knowledge, no gold standard exists to define the validity of insecticide-treated net. The WHOPES protocol does not report the initial choice of the number of 20 mosquitoes per LLIN side, neither the choice of 80% mortality nor 95% KD cutoffs for determining a LLIN as valid. These values were likely chosen arbitrarily.

The 95% CIs of the error in the measure of the proportion of valid LLINs was measured by resampling 10,000 times a database for sample sizes for nets varying between 20 and 160 LLINs, by increments of 20 units. The minimum sample size required to achieve a precision of ±5 pp was defined as the sample size of LLINs above which the upper limit of the 95% CI of the error in the measure of the proportion of valid LLINs did not exceed +5 pp and the lower limit was not inferior to 5 pp.

## Electronic supplementary material

Supplementary information

## References

[1] WHO. World malaria report 2013 (World Health Organization, Geneva, 2014).

[2] Tungu P (2010). Evaluation of PermaNet 3.0 a deltamethrin-PBO combination net against *Anopheles gambiae* and pyrethroid resistant *Culex quinquefasciatus* mosquitoes: an experimental hut trial in Tanzania. Malar. J..

[3] WHO. Guidelines for laboratory and field-testing of long-lasting insecticidal nets (World Health Organization, Geneva, 2013).

[4] Mittal PK, Sood RD, Kapoor N, Razdan RK, Dash AP (2012). Field evaluation of Icon®Life, a long-lasting insecticidal net (LLIN) against *Anopheles culicifacie*s and transmission of malaria in District Gautam Budh Nagar (Uttar Pradesh), Indi*a*. J. Vector Borne Dis..

[5] Randriamaherijaona S, Raharinjatovo J, Boyer S (2017). Durability monitoring of long-lasting insecticidal (mosquito) nets (LLINs) in Madagascar: physical integrity and insecticidal activity. Malar. J..

[6] Okumu FO (2012). Implications of bio-efficacy and persistence of insecticides when indoor residual spraying and long-lasting insecticide nets are combined for malaria prevention. Malar. J..

[7] Okia M (2013). Bioefficacy of long-lasting insecticidal nets against pyrethroid-resistant populations of *Anopheles gambiae s*.*s*. from different malaria transmission zones in Uganda. Parasit. Vectors..

[8] Yewhalaw D (2012). Bioefficacy of selected long-lasting insecticidal nets against pyrethroid resistant *Anopheles arabiensis* from south-western Ethiopia. Parasit. Vectors..

[9] Bhatt RM, Sharma SN, Uragayala S, Dash AP, Kamaraju R (2012). Effectiveness and durability of Interceptor® long-lasting insecticidal nets in a malaria endemic area of central India. Malar. J..

[10] Mattern C (2016). “Tazomoka Is Not a Problem”. Local Perspectives on Malaria, Fever Case Management and Bed Net Use in Madagascar. PLoS One..

[11] Randriamaherijaona S, Velonirina HJ, Boyer S (2016). Susceptibility status of A*nopheles arabiensis* (Diptera: Culicidae) commonly used as biological materials for evaluations of malaria vector control tools in Madagascar. Malar. J..

[12] Cornel, A. J. & Collins, S. H. *PCR of the ribosomal DNA intergenic spacer regions as a method for identifying mosquitoes in the Anopheles gambiae* complex in *Species Diagnostic Protocols*. 321–332 (Springer, 1996). 10.1385/0-89603-323-6:321 8751368

[13] Martinez Torres D (1998). Molecular characterization of pyrethroid knockdown resistance (kdr) in the major malaria vector *Anopheles gambiae s*.*s*. Insect Molec. Biol..

[14] Weill M (2004). The unique mutation in *ace-1* giving high insecticide resistance is easily detectable in mosquito vectors. Insect Molec. Biol..

