# Supplementary material for: Testing bio-efficacy of insecticide-treated nets with fewer mosquitoes for enhanced malaria control
Source: Sci Rep. 2018 Nov 13;8:16769. doi: 10.1038/s41598-018-34979-3 (PMC6233220; doi:10.1038/s41598-018-34979-3)
Supplement: Supplementary file 1 — Supplementary information [file 41598_2018_34979_MOESM1_ESM.docx]

**Supplementary information**

# Title

Testing bio-efficacy of insecticide-treated nets with fewer mosquitoes for enhanced malaria control

# Author list

Sebastien Boyer^1,2*^, Emilie Pothin^3^, Sanjiarizaha Randriamaherijaona^1,4^, Christophe Rogier^5,6,7^, Thomas Kesteman^5,8^

# Authors affiliations

^1^ Unité d’Entomologie Médicale, Institut Pasteur de Madagascar, BP 1274 Avaradoha, Antananarivo 101, Madagascar

^2^ Medical Entomology Platform, Institut Pasteur du Cambodge, 5 Boulevard Monivong, Phnom Penh, Cambodia

^3^ Department of Epidemiology and Public Health, Swiss Tropical and Public Health Institute, Basel, Switzerland.

^4^ Ecole doctorale Sciences de la vie et de l’environnement, Université d’Antananarivo, Antananarivo 101, Madagascar

^5^ Malaria Research Unit, Institut Pasteur de Madagascar, BP 1274 Avaradoha, Antananarivo 101, Madagascar

^6^ Unité de recherche sur les maladies infectieuses et tropicales émergentes (URMITE) - UMR 6236, 27 boulevard Jean Moulin, 13385 Marseille Cedex 05, France

^7^ Institute for Biomedical Research of the French Armed Forces (IRBA), BP 73, 91223 Brétigny-Sur-Orge Cedex, France

^8^ Fondation Mérieux, 17 rue Bourgelat, 69002 Lyon, France

## Competing Interests statement

The author(s) declare no competing financial and non-financial interests.

## List of Annexes

Annex 1: Validity, mortality and knock-down (KD) rates by brand

Annex 2: 95 and 99 confidence intervals of the precision in the measure of the proportion of valid LLINs

Annex 3: R code of trade-off analysis between efforts and accuracy

Annex 4: Bayesian model for variation in the outcomes

Annex 5: WinBUGS code

**Supplementary Dataset**

## Annex 1: Analysis by brand

| Brand | Insecticide | N | % valid LLIN | Range mortality per LLIN | Range KD per LLIN |
| --- | --- | --- | --- | --- | --- |
| **A** | α-cypermethrin | 80 | 45.0 | 14 – 100% | 34 – 100% |
| **B** | Deltamethrin | 57 | 19.3 | 11 – 100% | 25 – 100% |
| **C** | Deltamethrin | 98 | 30.6 | 2 – 100% | 17 – 100% |

## Annex 2: Confidence intervals of the precision in the measure of the proportion of valid LLINs

### 95% Confidence Intervals

|  |  | N cones | | | |
| --- | --- | --- | --- | --- | --- |
|  | N LLINs | 1 | 2 | 3 | 4 |
| Mortality & Knock-Down | 20 | -6.2,14.89 | -8,7.86 | -8.57,5.13 | -5.89,5.72 |
|  | 40 | -3.43,11.35 | -5.62,5.44 | -6.4,3.17 | -4.01,3.69 |
|  | 60 | -2.23,9.84 | -4.65,4.33 | -5.44,2.27 | -3.15,2.82 |
|  | 80 | -1.45,8.92 | -4.04,3.72 | -4.85,1.75 | -2.62,2.28 |
|  | 100 | -0.93,8.4 | -3.64,3.33 | -4.46,1.38 | -2.21,1.89 |
|  | 120 | -0.54,7.91 | -3.33,2.98 | -4.16,1.12 | -1.89,1.54 |
|  | 140 | -0.24,7.59 | -3.09,2.74 | -3.93,0.89 | -1.62,1.27 |
|  | 160 | -0.03,7.26 | -2.9,2.55 | -3.76,0.7 | -1.38,1.03 |
| Mortality |  | 1 | 2 | 3 | 4 |
|  | 20 | -5,9.23 | -4.45,5.78 | -3.85,4.33 | -3.24,3.16 |
|  | 40 | -3.19,6.67 | -2.95,4.07 | -2.56,2.92 | -2.15,2.08 |
|  | 60 | -2.41,5.54 | -2.32,3.31 | -2.03,2.4 | -1.64,1.61 |
|  | 80 | -1.92,4.93 | -1.94,2.89 | -1.68,2.04 | -1.34,1.3 |
|  | 100 | -1.57,4.56 | -1.69,2.6 | -1.47,1.82 | -1.12,1.08 |
|  | 120 | -1.32,4.25 | -1.5,2.39 | -1.31,1.64 | -0.94,0.91 |
|  | 140 | -1.14,3.99 | -1.37,2.24 | -1.19,1.52 | -0.79,0.77 |
|  | 160 | -0.97,3.81 | -1.26,2.1 | -1.07,1.41 | -0.66,0.64 |
| Knock-Down |  | 1 | 2 | 3 | 4 |
|  | 20 | -6.39,16.7 | -8.86,8.71 | -9.64,5.37 | -5.79,6.89 |
|  | 40 | -3.37,12.96 | -6.28,5.99 | -7.29,3.21 | -3.8,4.67 |
|  | 60 | -1.99,11.3 | -5.17,4.81 | -6.27,2.19 | -2.87,3.7 |
|  | 80 | -1.13,10.26 | -4.52,4.12 | -5.66,1.61 | -2.31,3.07 |
|  | 100 | -0.56,9.66 | -4.08,3.64 | -5.22,1.21 | -1.85,2.63 |
|  | 120 | -0.14,9.17 | -3.72,3.31 | -4.9,0.9 | -1.5,2.26 |
|  | 140 | 0.19,8.82 | -3.45,3.02 | -4.62,0.66 | -1.21,1.96 |
|  | 160 | 0.5,8.49 | -3.23,2.79 | -4.44,0.46 | -0.93,1.69 |

### 99% Confidence Intervals

|  |  | N cones | | | | |
| --- | --- | --- | --- | --- | --- | --- |
|  | N LLINs | 1 | 2 | 3 | 4 |  |
| Mortality & Knock-Down | 20 | -9.18,19.11 | -10.64,10.88 | -11.05,7.4 | -7.8,7.65 |  |
|  | 40 | -5.51,14.1 | -7.46,7.36 | -8.03,4.66 | -5.21,4.93 |  |
|  | 60 | -3.93,11.93 | -6,5.88 | -6.71,3.52 | -4.05,3.75 |  |
|  | 80 | -2.94,10.77 | -5.22,5 | -5.94,2.79 | -3.34,3.03 |  |
|  | 100 | -2.27,10 | -4.67,4.49 | -5.39,2.3 | -2.81,2.51 |  |
|  | 120 | -1.78,9.29 | -4.29,4.02 | -4.99,1.96 | -2.4,2.05 |  |
|  | 140 | -1.4,8.84 | -3.97,3.73 | -4.65,1.66 | -2.07,1.71 |  |
|  | 160 | -1.09,8.5 | -3.71,3.42 | -4.4,1.44 | -1.74,1.39 |  |
| Mortality |  | 1 | 2 | 3 | 4 |  |
|  | 20 | -7.69,12.59 | -6.51,8.14 | -5.54,6.06 | -4.4,4.27 |  |
|  | 40 | -4.68,8.7 | -4.13,5.45 | -3.52,4 | -2.85,2.76 |  |
|  | 60 | -3.59,7.1 | -3.26,4.43 | -2.78,3.19 | -2.13,2.08 |  |
|  | 80 | -2.94,6.2 | -2.69,3.8 | -2.32,2.7 | -1.72,1.69 |  |
|  | 100 | -2.45,5.69 | -2.37,3.4 | -2,2.39 | -1.44,1.41 |  |
|  | 120 | -2.15,5.21 | -2.09,3.1 | -1.8,2.15 | -1.21,1.18 |  |
|  | 140 | -1.88,4.91 | -1.93,2.87 | -1.63,1.96 | -1.01,1 |  |
|  | 160 | -1.68,4.64 | -1.76,2.72 | -1.48,1.83 | -0.85,0.83 |  |
| Knock-Down |  | 1 | 2 | 3 | 4 |  |
|  | 20 | -9.67,21.16 | -11.8,11.88 | -12.34,7.87 | -7.82,9.02 |  |
|  | 40 | -5.69,15.92 | -8.22,7.99 | -9.05,4.87 | -5.1,6.02 |  |
|  | 60 | -3.94,13.6 | -6.68,6.45 | -7.61,3.63 | -3.86,4.7 |  |
|  | 80 | -2.78,12.27 | -5.78,5.58 | -6.8,2.85 | -3.12,3.91 |  |
|  | 100 | -2.09,11.46 | -5.26,4.93 | -6.23,2.22 | -2.52,3.32 |  |
|  | 120 | -1.48,10.66 | -4.78,4.44 | -5.77,1.81 | -2.07,2.82 |  |
|  | 140 | -1.1,10.24 | -4.41,4.1 | -5.4,1.53 | -1.7,2.43 |  |
|  | 160 | -0.68,9.82 | -4.16,3.77 | -5.16,1.27 | -1.33,2.09 |  |

## Annex 3: R code of trade-off analysis between efforts and accuracy

### Trade-off between efforts and specificity/sensitivity ###

# function to find limits (lower & upper) of confidence interval

fnToFindLimitCI = function(x,n,cutoff,GoodFail) {

pbinom(round(n*cutoff,0)-1, n, x, lower.tail = GoodFail) - 0.1 #0.1 = probability to classify erroneously the net

}

# Mortality rate

cat("1. Mortality rate","\n")

Cutoff = 0.80

for ( n_mosquito in c(25,50,75,100)) { #for 1-4 cones

cat(paste("N cones : ",n_mosquito/25,"\n"))

ll = uniroot(fnToFindLimitCI, c(0, 1), n=n_mosquito,cutoff=Cutoff,GoodFail=F)$root #lower limit

ul = uniroot(fnToFindLimitCI, c(0, 1), n=n_mosquito,cutoff=Cutoff,GoodFail=T)$root #upper limit

cat(paste("Interval =",round(ll*100,1),"to",round(ul*100,1),". Range =",round(abs(ll-ul)*100,1),"\n"))

}

# Knock-down rate

cat("2. Knock-down rate","\n")

Cutoff = 0.95

for ( n_mosquito in c(25,50,75,100)) { #for 1-4 cones

cat(paste("N cones : ",n_mosquito/25,"\n"))

ll = uniroot(fnToFindLimitCI, c(0, 1), n=n_mosquito,cutoff=Cutoff,GoodFail=F)$root #lower limit

ul = uniroot(fnToFindLimitCI, c(0, 1), n=n_mosquito,cutoff=Cutoff,GoodFail=T)$root #upper limit

cat(paste("Interval =",round(ll*100,1),"to",round(ul*100,1),". Range =",round(abs(ll-ul)*100,1),"\n"))

}

## Annex 4: Bayesian model for variation in the outcomes

Let $X_{i,j,k}$ and $Y_{i,j,k}$ respectively be the number of mosquitoes knocked down after 1h and killed after 24h for mosquito net $i$, on side j and in cone $k$. These counts are assumed to be binomially distributed as follows:

$\left\{ \begin{aligned} X_{i,j,k}\sim Bin(\mu_{i,j},n_{i,j,k}) \\ Y_{i,j,k}\sim Bin(\eta_{i,j},n_{i,j,k}) \end{aligned} \right.$, (1)

where $\mu_{i,j}$ and $\eta_{i,j}$ are the probabilities of being knocked down after 1h and killed after 24h respectively, and $n_{i,j,k}$ is the total number of mosquitoes tested for each cone, i.e. equal to 5.

We assume a Bayesian framework where these probabilities have distributions as follows:

$\left\{ \begin{aligned} logit\left( \mu_{i,j} \right)={logit(p}_{i})+\alpha_{i,j} \\ logit\left( \eta_{i,j} \right)={logit(q}_{i})+\delta_{i,j} \end{aligned} \right.$ (2)

Where $p_{i}$, is the average knock-down probability for the net, $q_{i}$, is the corresponding mortality and $\alpha_{i,j}$ and $\delta_{i,j}$ represent the effects of the specific side of the net. The former two parameters are assumed to vary by net around population averages, so that:

$\left\{ \begin{aligned} logit\left( p_{i} \right)\sim N(\mu_{0},\sigma_{0}^{2}) \\ logit\left( q_{i} \right)\sim N(\mu_{1},\sigma_{1}^{2}) \end{aligned} \right.$ (3)

While the effects of the side of the net are centered around zero:

$\left\{ \begin{aligned} \alpha_{i,.}\sim N(0,\sigma_{2}^{2}) \\ \delta_{i,.}\sim N(0,\sigma_{3}^{2}) \end{aligned} \right.$ (4)

With prior distributions for the hyperparameters:

$\mu_{x\in[0,3]}, \sigma_{x\in[0,3]}$ $\forall x, \mu_{x}\sim N\left( 0,{10}^{-3} \right)\mathrm{and}\sigma_{x}\sim U(0,10)$

A Markov chain Monte Carlo algorithm was used to estimate the various parameters in the program WinBUGS[1].

### Reference

[1] D. J. Lunn, A. Thomas, N. Best, and D. Spiegelhalter, “WinBUGS - A Bayesian modelling framework: Concepts, structure, and extensibility,” *Stat. Comput.*, vol. 10, no. 4, pp. 325–337, 2000.

## Annex 5: R code for WinBUGS program (JAG scripts)

#libraries

library(reshape)

library(R2WinBUGS)

library(R2jags)

library(rjags)

#=====================================================================######

######################################### Mortality

######

#=====================================================================

model_nets_mortality.mod <- function(){

## for each row (cone per facet per mosquito net)

for( i in 1 : N_rows) {

#tot[i] <- dead[i] + alive[i]

## mortality - dead=> number of dead mosquitoes after 24h, mu=> mortality rate per cone

dead[i] ~ dbin(mu[i],tot)

logit(mu[i]) <- logitmu[net[i]] + alphasmu[net[i],np[i]]

}

## for each mosquito net

for( i1 in 1 : N_net) {

#mui1: mortality rate

logitmu[i1] ~ dnorm(mu1 , tau1)

mui1[i1] <- 1/(1+ exp(-logitmu[i1]))

### Failure rate - the mean value for each iteration will provide the pobability of the mortality rate to be above 80% (i.e proba of the net to be valid)

failure_mu[i1] <- 1 -step(mui1[i1]-Threshold_mortality)

failure[i1]<-failure_mu[i1]

for( side in 1 : N_side) {

alphasmu[i1,side] ~ dnorm(0 , tau2)

}

#total number of dead mosquitoes per net

for(i in 1:N_rows){

temp_tot_dead[i,i1]<-dead[i]*equals(net[i],i1)

}

tot_dead[i1]<-sum(temp_tot_dead[,i1])

obs_failure[i1]<-1-step(tot_dead[i1]-Threshold_dead)

PP[i1]<-(1-failure[i1])*(1-obs_failure[i1])

FP[i1]<- failure[i1]*(1-obs_failure[i1])

NN[i1]<-failure[i1]*obs_failure[i1]

FN[i1]<-(1-failure[i1])*obs_failure[i1]

}

proba_PP <- mean(PP[])

proba_FP <- mean(FP[])

proba_NN <- mean(NN[])

proba_FN <- mean(FN[])

True_pos<-proba_PP+proba_FN

True_neg<-proba_NN+proba_FP

#Se<-proba_PP/True_pos

#Specificity<-proba_NN/(proba_NN+proba_FP)

## Define priors

sigma0~ dunif(0,10)

sigma1~ dunif(0,10)

sigma2~ dunif(0,10)

tau0<-1/(sigma0*sigma0)

tau1<-1/(sigma1*sigma1)

tau2<-1/(sigma2*sigma2)

mu1 ~ dnorm(0.0,1.0E-3)

}

#=====================================================================######

######################################### Knock Down

######

#=====================================================================

model_nets_kd.mod <- function(){

## for each row (cone per facet per mosquito net)

for( i in 1 : N_rows) {

## KD- dead=> number of dead mosquitoes after 24h, mu=> KDrate per cone

kdn[i] ~ dbin(kd[i],tot)

logit(kd[i]) <- logitkd[net[i]] + alphaskd[net[i],np[i]]

}

## for each mosquito net

for( i1 in 1 : N_net) {

#mui1: KDrate

logitkd[i1] ~ dnorm(mu1 , tau1)

kdi1[i1] <- 1/(1+ exp(-logitkd[i1]))

### Failure rate - the mean value for each iteration will provide the pobability of the KDrate to be above 80% (i.e proba of the net to be valid)

failure[i1] <- 1 -step(kdi1[i1]-Threshold_rate)

for( side in 1 : N_side) {

alphaskd[i1,side] ~ dnorm(0 , tau2)

}

#total number of dead mosquitoes per net

for(i in 1:N_rows){

temp_tot_kd[i,i1]<-kdn[i]*equals(net[i],i1)

}

tot_kd[i1]<-sum(temp_tot_kd[,i1])

obs_failure[i1]<-1-step(tot_kd[i1]-Threshold_number)

PP[i1]<-(1-failure[i1])*(1-obs_failure[i1])

FP[i1]<- failure[i1]*(1-obs_failure[i1])

NN[i1]<-failure[i1]*obs_failure[i1]

FN[i1]<-(1-failure[i1])*obs_failure[i1]

}

proba_PP <- mean(PP[])

proba_FP <- mean(FP[])

proba_NN <- mean(NN[])

proba_FN <- mean(FN[])

True_pos<-proba_PP+proba_FN

True_neg<-proba_NN+proba_FP

## Define priors

sigma0~ dunif(0,10)

sigma1~ dunif(0,10)

sigma2~ dunif(0,10)

tau0<-1/(sigma0*sigma0)

tau1<-1/(sigma1*sigma1)

tau2<-1/(sigma2*sigma2)

mu1 ~ dnorm(0.0,1.0E-3)

}

#=====================================================================######

######################################### Knock Down

######

#=====================================================================

#=====================================================================######

######################################### Mortality & Knock Down

######

#=====================================================================

model_nets.mod <- function(){

## for each row (cone per facet per mosquito net)

for( i in 1 : N_rows) {

#tot[i] <- dead[i] + alive[i]

## KD- dead=> number of dead mosquitoes after 24h, mu=> KDrate per cone

dead[i] ~ dbin(mu[i],tot)

logit(mu[i]) <- logitmu[net[i]] + alphasmu[net[i],np[i]]

kdn[i] ~ dbin(kd[i],tot)

logit(kd[i]) <- logitkd[net[i]] + alphaskd[net[i],np[i]]

}

## for each mosquito net

for( i1 in 1 : N_net) {

#mui1: KDrate

logitkd[i1] ~ dnorm(mu1 , tau1)

kdi1[i1] <- 1/(1+ exp(-logitkd[i1]))

### Failure rate - the mean value for each iteration will provide the pobability of the KDrate to be above 80% (i.e proba of the net to be valid)

failure_kd[i1] <- 1 -step(kdi1[i1]-Threshold_rate_kd)

logitmu[i1] ~ dnorm(mu0 , tau0)

mui1[i1] <- 1/(1+ exp(-logitmu[i1]))

failure_mu[i1] <- 1 -step(mui1[i1]-Threshold_rate_mortality)

failure[i1] <- failure_mu[i1]*failure_kd[i1]

for( side in 1 : N_side) {

alphasmu[i1,side] ~ dnorm(0 , tau2)

alphaskd[i1,side] ~ dnorm(0 , tau3)

}

#total number of dead mosquitoes per net

for(i in 1:N_rows){

temp_tot_kd[i,i1]<-kdn[i]*equals(net[i],i1)

temp_tot_mortality[i,i1]<-dead[i]*equals(net[i],i1)

}

tot_kd[i1]<-sum(temp_tot_kd[,i1])

tot_mortality[i1]<-sum(temp_tot_mortality[,i1])

obs_failure_kd[i1]<-1-step(tot_kd[i1]-Threshold_number_kd)

obs_failure_mortality[i1]<-1-step(tot_mortality[i1]-Threshold_number_mortality)

obs_failure[i1]<-obs_failure_mortality[i1]*obs_failure_kd[i1]

PP[i1]<-(1-failure[i1])*(1-obs_failure[i1])

FP[i1]<- failure[i1]*(1-obs_failure[i1])

NN[i1]<-failure[i1]*obs_failure[i1]

FN[i1]<-(1-failure[i1])*obs_failure[i1]

}

proba_PP <- mean(PP[])

proba_FP <- mean(FP[])

proba_NN <- mean(NN[])

proba_FN <- mean(FN[])

True_pos<-proba_PP+proba_FN

True_neg<-proba_NN+proba_FP

## Define priors

sigma0~ dunif(0,10)

sigma1~ dunif(0,10)

sigma2~ dunif(0,10)

sigma3~ dunif(0,10)

tau0<-1/(sigma0*sigma0)

tau1<-1/(sigma1*sigma1)

tau2<-1/(sigma2*sigma2)

tau3<-1/(sigma2*sigma2)

mu0 ~ dnorm(0.0,1.0E-3)

mu1 ~ dnorm(0.0,1.0E-3)

}
